# 188. Opportunities for antimicrobial stewardship: Time to optimal therapy and vancomycin use in gram-positive and contaminated blood cultures before and after implementation of rapid blood culture diagnostic testing

**DOI:** 10.1093/ofid/ofac492.266

**Published:** 2022-12-15

**Authors:** Kelsey Olion, Laura Hamann, Rebecca Jennemann, Julie A Shapiro-Schere, Yvonne J Burnett

**Affiliations:** Missouri Baptist Medical Center, Saint Louis, Missouri; Missouri Baptist Medical Center, Saint Louis, Missouri; Missouri Baptist Medical Center, Saint Louis, Missouri; Northwest Infectious Disease, Bridgeton, Missouri; University of Health Sciences and Pharmacy in St. Louis, St. Louis, Missouri

## Abstract

**Background:**

Rapid molecular diagnostic tests improve time to organism identification and may expedite antimicrobial de-escalation. Through faster detection of contaminated samples, rapid blood culture identification (BCID) should also reduce unnecessary antibiotic use. BioFire FilmArray BCID was implemented in July 2019 at a community hospital. This study compared antimicrobial use for gram-positive blood cultures before and after BCID implementation to identify areas for further antimicrobial stewardship intervention.

**Methods:**

This retrospective cohort study evaluated adult patients at a 450-bed community hospital with gram-positive blood culture results between February and June 2018 and 2019 (pre-and post-BCID). Patients were excluded for polymicrobial bacteremia, pregnancy, or expiration/hospice status before blood culture positivity. The primary outcome was time to optimal therapy (TTOT) for bacteremia, defined as time to antimicrobial de-escalation, discontinuation, or result for untreated contaminated cultures. Time to organism identification, receipt of optimal therapy, and vancomycin duration were also evaluated, in addition to subgroup analysis of contaminated cultures (identified by microbiology comment or physician note).

**Results:**

Of the 232 patients, median age was 70 years and 54.3% were male. Coagulase-negative staphylococci was most commonly isolated (n=104, 45%) and contamination rate was 53.4%. Median TTOT improved from 53.3 vs 28 hours, *p*< 0.001 in pre- vs post-groups, respectively, and optimal therapy selection increased from 83% to 93%, *p*=0.028. Median time to organism identification was 60.2 vs 23.5 hours, p< 0.001. Median duration for vancomycin (35.1 vs 25.2 hours, *p*=0.231) and overall antimicrobial duration did not differ between groups. Among contaminated blood cultures, TTOT improved (54 vs 27.3 hours, *p*< 0.001) and vancomycin median duration decreased (27.5 vs 16.6 hours, *p*=0.253).

**Conclusion:**

Rapid BCID significantly improved TTOT, resulting in improved rates of optimal therapy and an observed reduced vancomycin duration. High rates of contaminated blood cultures identified opportunities for coordination with antimicrobial stewardship to reduce contamination rates and improve antimicrobial use.

**Disclosures:**

**All Authors**: No reported disclosures.

